# New climatic targets against global warming: will the maximum 2 °C temperature rise affect estuarine benthic communities?

**DOI:** 10.1038/s41598-017-04309-0

**Published:** 2017-06-20

**Authors:** Daniel Crespo, Tiago Fernandes Grilo, Joana Baptista, João Pedro Coelho, Ana Isabel Lillebø, Fernanda Cássio, Isabel Fernandes, Cláudia Pascoal, Miguel Ângelo Pardal, Marina Dolbeth

**Affiliations:** 1 Centre for Functional Ecology - CFE, Department of Life Sciences, University of Coimbra, Calçada Martim de Freitas, 3000-456 Coimbra, Portugal; 2 MARE - Marine and Environmental Sciences Centre, Laboratório Marítimo da Guia - Faculdade de Ciências da Universidade de Lisboa, Av. Nossa Senhora do Cabo, 939, 2750-374 Cascais, Portugal; 3 CIIMAR - Interdisciplinary Centre of Marine and Environmental Research, Novo Edifício do Terminal de Cruzeiros do Porto de Leixões, Avenida General Norton de Matos s/n, 4450-208 Matosinhos, Portugal; 4 Department of Chemistry & CESAM, University of Aveiro, Campus Universitário de Santiago, 3810-193 Aveiro, Portugal; 5 Department of Biology & CESAM, University of Aveiro, Campus Universitário de Santiago, 3810-193 Aveiro, Portugal; 6 Centre of Molecular and Environmental Biology (CBMA), Department of Biology, University of Minho, Campus de Gualtar, 4710-057 Braga, Portugal; 7 Institute of Science and Innovation for Bio-sustainability (IB-S), University of Minho, Campus de Gualtar, 4710-057 Braga, Portugal

## Abstract

The Paris Agreement signed by 195 countries in 2015 sets out a global action plan to avoid dangerous climate change by limiting global warming to remain below 2 °C. Under that premise, *in situ* experiments were run to test the effects of 2 °C temperature increase on the benthic communities in a seagrass bed and adjacent bare sediment, from a temperate European estuary. Temperature was artificially increased *in situ* and diversity and ecosystem functioning components measured after 10 and 30 days. Despite some warmness effects on the analysed components, significant impacts were not verified on macro and microfauna structure, bioturbation or in the fluxes of nutrients. The effect of site/habitat seemed more important than the effects of the warmness, with the seagrass habitat providing more homogenous results and being less impacted by warmness than the adjacent bare sediment. The results reinforce that most ecological responses to global changes are context dependent and that ecosystem stability depends not only on biological diversity but also on the availability of different habitats and niches, highlighting the role of coastal wetlands. In the context of the Paris Agreement it seems that estuarine benthic ecosystems will be able to cope if global warming remains below 2 °C.

## Introduction

Despite specific adaptations to highly dynamic habitats such as estuaries, estuarine organisms can only cope with environmental variation within their specific range of tolerance. As such, species richness is generally low in estuaries, as few taxa evolved towards the required broad tolerance^1, 2^. Yet, estuaries are highly productive areas^3, 4^, due to continuous organic matter input from river basins, nutrient dynamics through decomposition and organic matter mineralization and habitat heterogeneity^4, 5^.

It has been accepted that biodiversity with all its components, from the microscopic to the landscape level, is fundamental for the ecosystem functioning^6–8^. For instance, biological and functional diversity are essential for a complementarity effect and functional redundancy in a system, i.e. different species with similar functions, which usually delivers a better functional performance towards environmental change^9–11^. Nevertheless, species diversity does not always cope with rapid ecosystem modification under external pressures^12, 13^, which are becoming more frequent in face of global ecological changes. In the short term, processes and functions can also depend on behavioural shifts^14^. Therefore, even within rich biological systems, ecosystem functions such as primary and secondary production, decomposition and nutrient cycling can be affected even without drastic changes in the community demographic figures. Multilevel faunal interactions (microfauna, meiofauna and macrofauna) represent an additional challenge for tracking flows of energy/biomass and nutrients.

*Ex situ* mesocosm experiments have proved to be efficient in measuring ecosystem functions and assessing the effect of changing diversity in those functions^14–16^ because control parameters can be easily tuned and models are straightforward to assess, with direct cause-effect responses^16–19^, although not without limitations. The interactive effects of multiple stressors on ecosystem functioning are difficult to predict, due to synergistic/antagonistic variation of complex natural systems^16^ or the effects of multifunctionality^9^. An experimental setup in field conditions is one way to account for more realistic responses and to understand how natural context may control biological responses (e.g. refs 19 and 20), by manipulating control/test variables, but keeping all the remaining natural variability (e.g. ref. 21). Nevertheless, it poses additional challenges in the experimental design and for the stabilization of control parameters^17, 22^.

Global changes induced by anthropogenic impact have increased severely during the 20^th^ century^23, 24^ and their outcome for biological systems are still unpredictable. However, in spite of the associated uncertainty, there is a general consensus regarding environmental and socio-economic implications derived from such changes^25, 26^. Several international agreements on greenhouse gases and other climate policies were assumed by major stakeholders in recent decade^27, 28^. However, an ambitious common goal regarding global warming was only assumed during the recent United Nations Conference on Climate Change, held in Paris, France, in 2015: to keep temperature rise below 2 °C, preferably 1.5 °C, above pre-industrial levels^29^. Therefore, it is highly relevant to measure responses of different functional groups within this new international framework. In fact, the Paris Conference on Climate Change appeals to the increase of scientific knowledge on the effects of climate, in order to support decision-making (article 7, paragraph 7 ref. 29). As such, the proposed research aims to contribute with science-based knowledge, focusing on the effect of a 2 °C temperature rise on benthic estuarine communities. For this, we performed a 30-day *in situ* experiment with benthic intertidal communities from a southern European temperate estuary, where a temperature increase was induced artificially. Data on micro and macrofauna diversity and important ecological processes (bioturbation) and functions (nutrient balance) were measured in order to contribute for a better knowledge about the effects of a mild temperature rise in the functioning of estuarine ecosystems. We expect changes in the benthic communities, namely decreased levels of diversity for both macrofauna and microfauna and changes in the dominant species under the effect of warmness, taking into account results from previous records on the effects of temperature increase in macrobenthic communities (e.g. refs 3 and 13). As for the ecosystem processes and functions, we also assume changes owing the differential communities expected under the warmness scenario. However, we cannot anticipate the change trajectory.

## Results

### Efficiency of the temperature increase

The warmness treatments were able to produce a differential temperature between the open boxes – “no warmness effect”, and the closed boxes – “warmness effect” (Supplementary Fig. S1). The temperature difference measured in the sediment at the daily peak was 2.35 °C for the sandflat and 1.52 °C for the *Zostera* bed (Supplementary Fig. S1), whose values were within the ones considered acceptable under the framework of the Paris United Nations Conference on Climate Change. Also, values for the continuous record (which includes high tides and night periods) denoted an average temperature rise on both sites (0.36 °C in the sandflat and 0.21 °C in the *Zostera* bed, Supplementary Fig. S1).

### Effect of the warmness treatment, habitat and time

#### Macrobenthic communities

**Figure 1:**
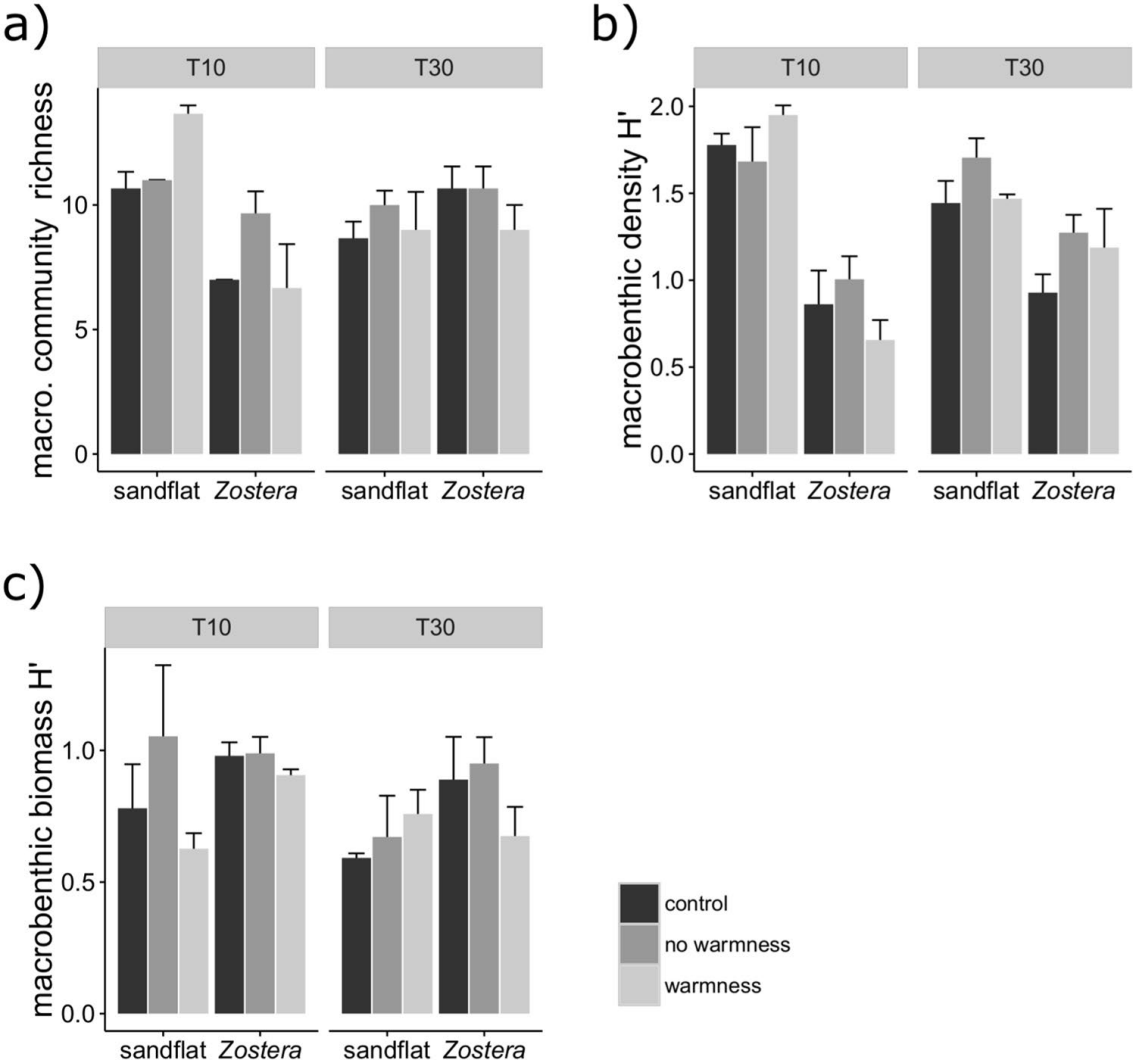
Mean + s.d. (n = 3) species richness (**a**) and Shannon-Wiener index of the macrobenthic communities measured with density (**b**) and biomass (**c**) in the sandflat and *Zostera* areas, under control, no warmness and warmness treatment, for the two time periods.

For the macrobenthic communities’ species richness, we found two significant interactions with site, in combination with time and in combination with the temperature treatment effect (Table 1). At T10 species richness was higher in the sandflat than in the *Zostera*, particularly for the “warmness” treatment (Fig. 1a, Table 1). However, at T30 the values were very similar between sites and treatments (Fig. 1a). The Shannon-Wiener index (H’) for macrobenthic density was higher in the sandflat for both times (Fig. 1b), with a significant interaction detected between site and time (Table 1). For the H’ estimated with biomass, only site became significant (Table 1), with the *Zostera* showing higher H’ values, except in the “no warmness” treatment, in T10 (Fig. 1c).

**Table 1:** Summary of significant terms from the 3-way PERMANOVA analyses, with macrobenthic community and respective diversity indices as dependent variables, and site, treatment and time as explanatory variables, with indication of the significant pairwise comparisons.

Dependent variable	Significant terms	d.f.	Pseudo-F	p-perm	Terms/levels of factor	p-perm
Species Richness	site × temperature	2	4.2065	0.021	warmness [sandflat vs *Zostera*]	0.021
	site × time	1	22.011	0.002	T10 [sandflat vs *Zostera*]	0.001
					Sandflat, Zostera: [T10 *vs* T30]	*<*0.015
Shannon (density)	site × time	1	12.761	0.002	T10, T30: [sandflat vs *Zostera*]	<0.004
					Sandflat, Zostera: [T10 *vs* T30]	<0.05
Shannon (biomass)	site	1	4.12	0.05	sandflat vs *Zostera*	0.05
Benthic community density	site × temperature	2	1.8469	0.037	Sandflat: [Control vs warmness]	0.056
					All treatments: [sandflat vs Zostera]	<0.05
	site × time	1	2.8878	0.01	For T10, T30: *Zostera* vs sandflat	0.001
					Sandflat, *Zostera*: T10 vs T30	<0.005
Benthic community biomass	site × temperature	2	2.171	0.032	Sandflat: [Control vs warmness]	0.017
					All treatments: [sandflat vs Zostera]	<0.05
	site × time	1	3.2585	0.019	For T10, T30: *Zostera* vs sandflat	<0.002
					Sandflat, Zostera: [T10 vs T30]	<0.04

**Figure 2:**
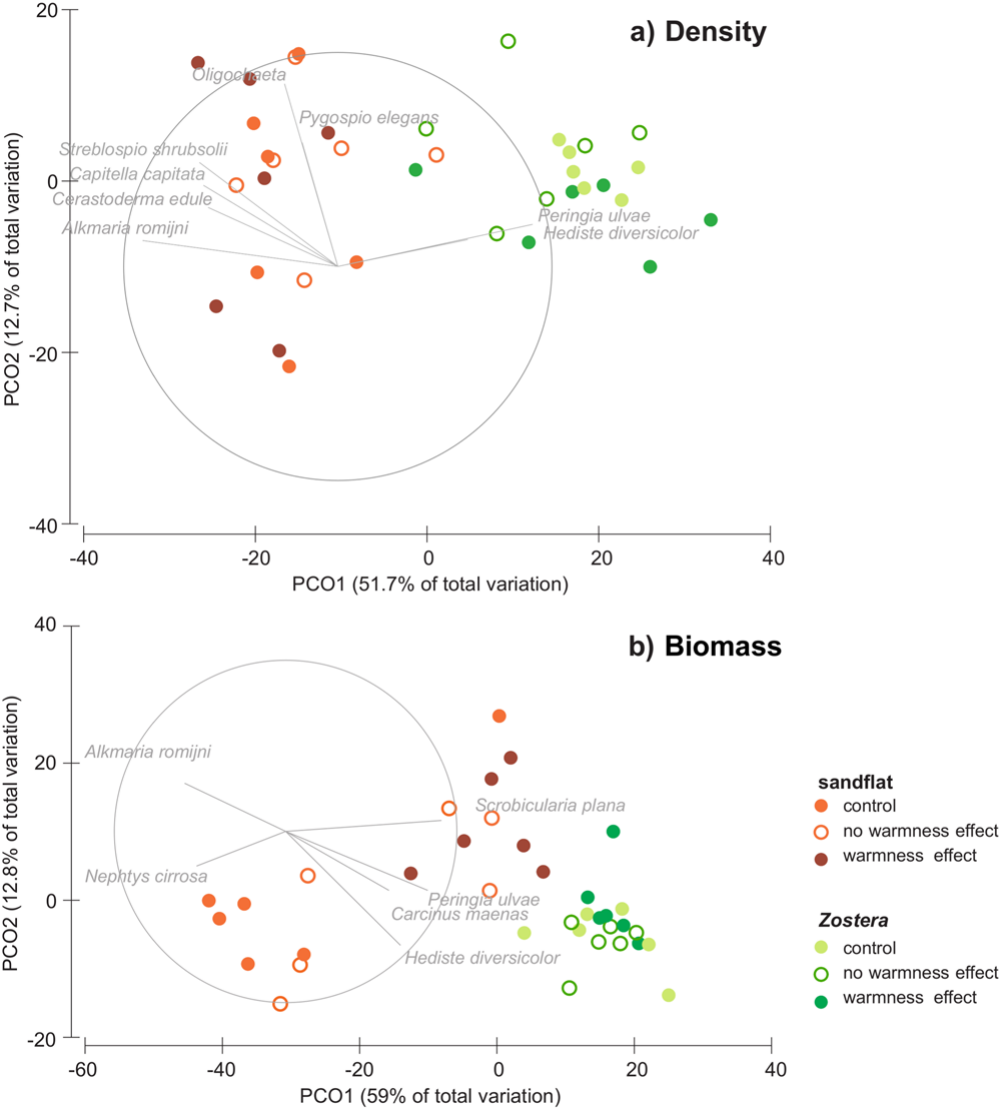
PCO ordination for macrobenthic community density and biomass for each area and treatment. The length and direction of each vector indicates the strength and sign of the relationship between species abundance or biomass and PCO axes, based on a Spearman correlation (only vector with length >0.5 were represented).

Regarding the macrobenthic community data, significant interactions between site and treatment and site and time were found (Table 1). However, differences were clearer per site, with *Peringia ulvae and Hediste diversicolor* associated with the seagrass, while small opportunist polychaetes and *Cerastoderma edule* were associated with the sandflat (Fig. 2a). Despite the significant interaction found between site and time, differences were significant for both time periods and sites, while for the treatment effect statistical differences were only found between control and the warmness treatment for the sandflat alone (Table 1).

For biomass data, we had similar results as the ones for density (two significant interactions, Table 1). Again, differences were very clear per site (Fig. 2b) and there were significant differences between T10 and T30 for the sandflat samples and when comparing sites within each time period (Table 1). Regarding the effect of temperature rise on macrobenthic fauna, results were clearer than with the density (Fig. 2b). However, PERMANOVA provided the same results: no statistical differences were found between control and the “no warmness effect” treatment, used here as a control for the box device, but there were significant differences between control *vs* “warmness effect” (Table 1, Fig. 2b). These differences seemed more evident for the sandflat (Fig. 2b), despite being significant for both sites (Table 1).

#### Microbial communities

**Figure 3:**
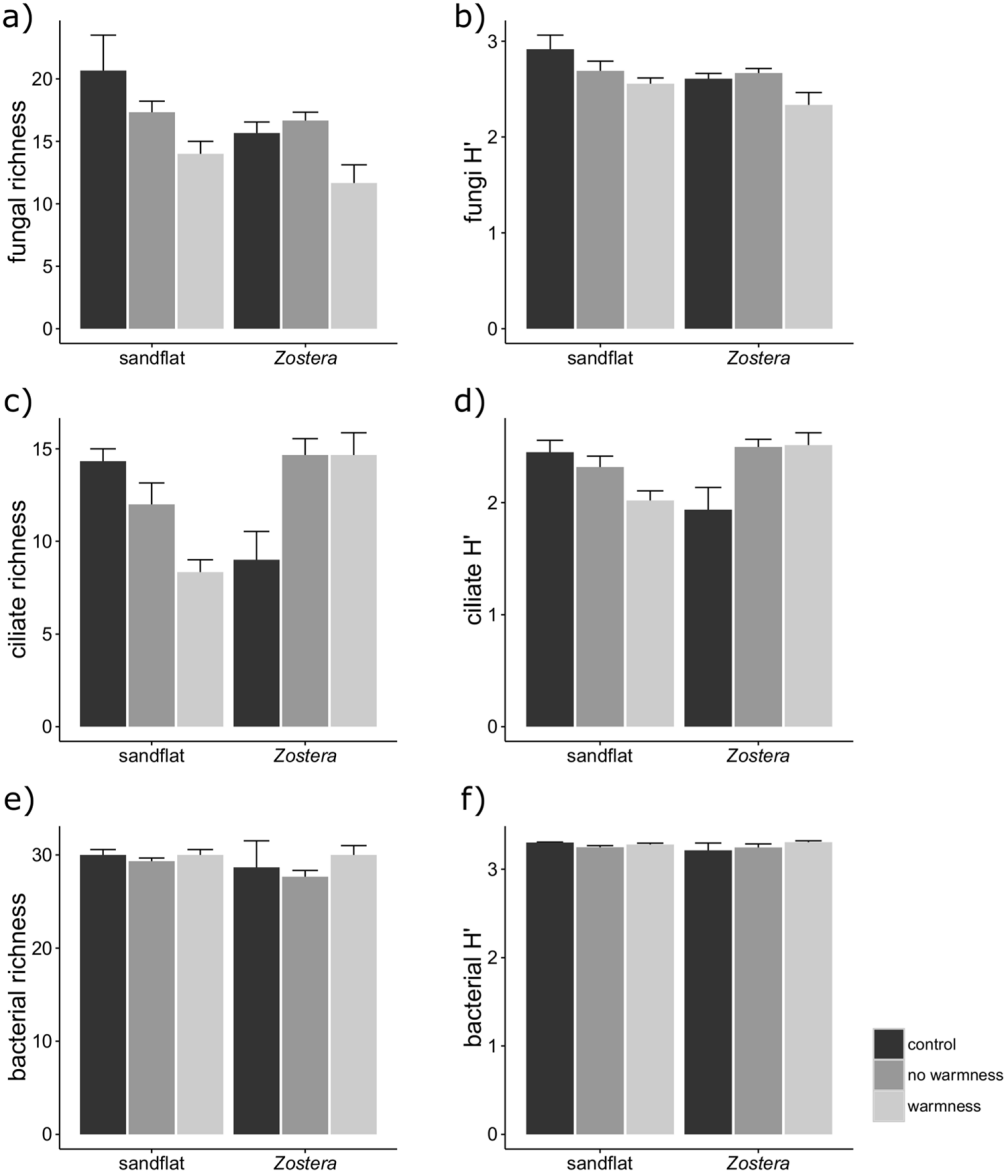
Mean + s.d. (n = 3) richness of fungal (**a**), ciliates (**c**) and bacterial (**e**) communities and Shannon-Wiener index of fungal (**b**), ciliates (**d**) and bacterial (**f**) communities on sediment samples from sandflat or *Zostera* under control, no warmness and warmness treatment.

Microfauna diversity and composition was only assessed in T10. A similar trend variation was observed for the fungal richness and Shannon-Wiener index, but with clearer differences among areas and treatments for the richness (Fig. 3a,b). Both indices were significantly higher in the sandflat area than in *Zostera*, with the highest values found on the control treatment associated with sandflat (Table 2, Fig. 3). Differences were also evident with regard to the warmness treatment effect (Table 2), particularly for the richness (Fig. 3a,b). Richness values were significantly lower in the “warmness” compared to the “no warmness” and control treatments (Tukey’s, p < 0.05), while Shannon-Wiener values were significantly lower in the “warmness” compared to the control (Tukey’s, p = 0.0182).

**Table 2:** Results from the 2-way ANOVA for the effect of site and temperature on fungal, ciliate and bacterial communities’ richness and Shannon-Wiener indices. For the bacterial Shannon-index a 2-way PERMANOVA was applied, as data did not pass the assumptions of parametric tests; ^a^- pseudo-F, ^b^- P(perm).

Dependent variable	Effect	S.S.	d.f.	M.S.	F-value	p-value
Fungal richness	temperature	94.33	2	47.17	7.13	0.009087
	site	32.00	1	32.00	4.84	0.048130
	temperature × site	14.33	2	7.17	1.08	0.369168
	error	79.33	12	6.61		
Fungi Shannon-Wiener index	temperature	0.32	2	0.16	5.63	0.018869
	site	0.153	1	0.15	5.30	0.040004
	temperature × site	0.06	2	0.03	1.11	0.360000
	error	0.35	12	0.03		
Ciliate richness	temperature	12.33	2	6.17	1.82	0.204069
	site	6.72	1	6.72	1.98	0.184389
	temperature × site	106.78	2	53.39	15.75	0.000440
	error	40.67	12	3.39		
Ciliate Shannon-Wiener index	temperature	0.14	2	0.07	1.70	0.225634
	site	0.01	1	0.01	0.31	0.588654
	temperature × site	0.80	2	0.40	9.50	0.003361
	error	0.50	12	0.04		
Bacterial richness	temperature	4.25 × 10^12^	2	2.13 × 10^12^	1.01	0.393082
	site	8.71 × 10^11^	1	8.71 × 10^11^	0.41	0.532230
	temperature × site	1.02 × 10^12^	2	5.12 × 10^11^	0.24	0.788013
	error	2.53 × 10^13^	12	2.11 × 10^12^		
Bacteria Shannon-Wiener index	temperature	2.11E-03	1	2.11E-03	0.46085^a^	0.558^b^
	site	6.69E-03	2	3.34E-03	0.73183^a^	0.524^b^
	temperature × site	1.04E-02	2	5.19E-03	1.1361^a^	0.367^b^
	Residual	5.48E-02	12	4.57E-03		

The trend variation in the diversity indices of the ciliate communities was also similar, but again, differences were clearer with richness (Fig. 3c,d). For both indices, the interaction between site and temperature was significant (Table 2). For the sandflat, both indices were highest in the control treatment and lowest in the “warmness” treatment (Fig. 3c,d). However, this trend was the opposite for the *Zostera*, which had the lowest values in the control treatment compared to the others (Fig. 3e,f). The same pattern was observed for the Shannon index (2-way ANOVA, p = 0.003; Table 2; Fig. 3d), with temperature treatment decreasing ciliates communities’ evenness in sandflat, but increasing ciliates communities’ evenness in *Zostera*.

Bacterial richness and Shannon-Wiener index varied within similar values for both sites and treatments (Fig. 3e,f), with no significant differences detected among factors (Table 2). Bacterial communities contained on average 30 species (Fig. 3e).

**Figure 4:**
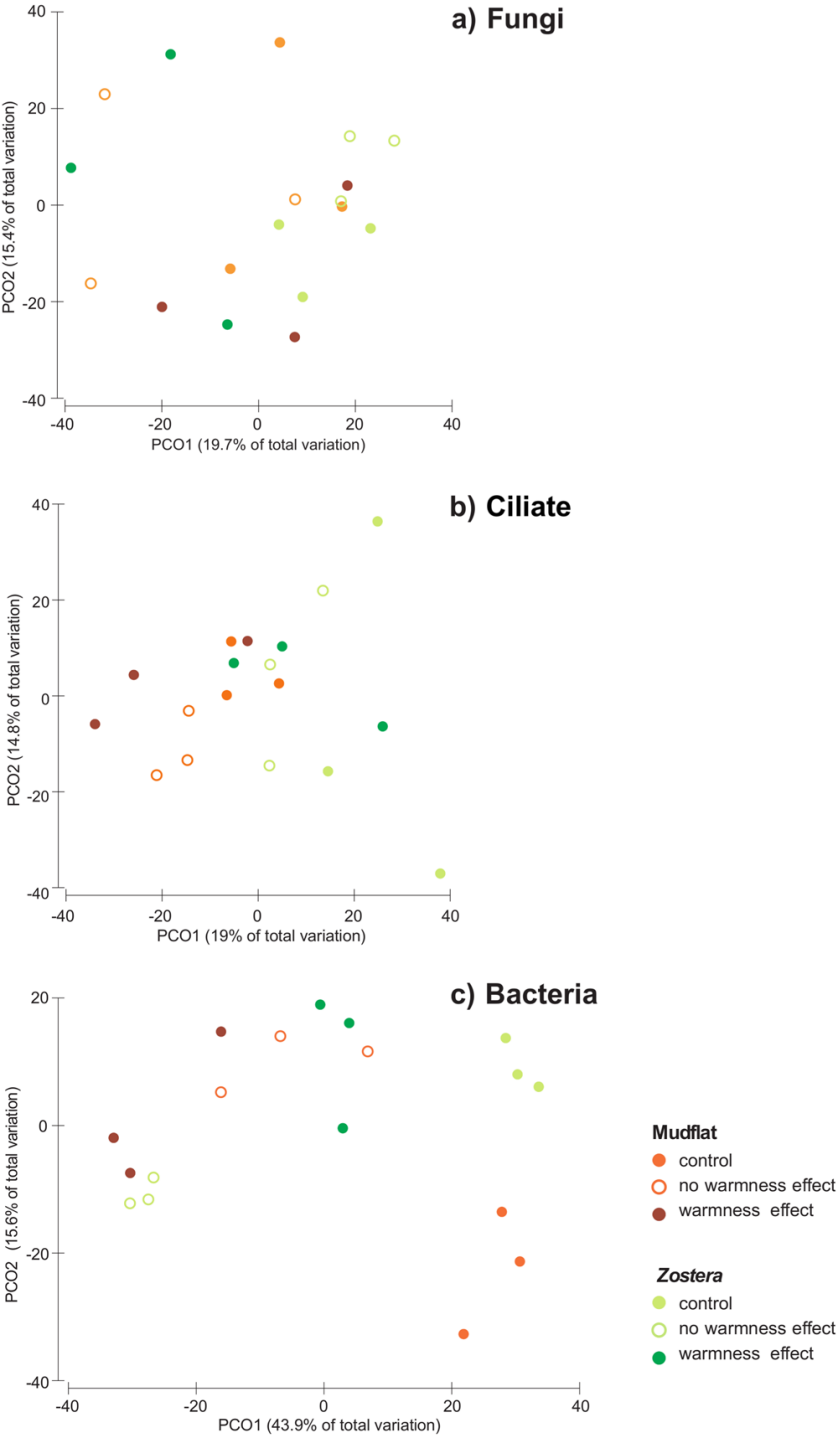
PCO ordination for fungal (**a**), ciliates (**b**) and bacterial (**c**) communities for each site and temperature treatment.

Regarding the composition and structure of the fungal, ciliate and bacterial communities, significant interactions were found between site and temperature for all communities (Table 3). However, the pairwise comparisons were not significant for any of the terms/factors pairs (Table 3). As such, we have considered the significance among main terms, which were always significant (Table 3). Despite this result, the variability explained in the PCO plots for the fungal and ciliate communities was relatively low (<25.1%) and the discrimination among factors was not that clear (Fig. 4a,b). In both these communities, significant differences were found per site and among the control with the other treatments (Table 3). For the bacterial communities, statistical differences were found per site and among all temperature treatments (Table 3). The variability explained in the PCO plot was higher than for the other community (about 60%), with the control samples clearly separated from the other treatments (Fig. 4c). For the *Zostera*, differences among treatments were also clearer (Fig. 4c).

**Table 3:** Summary of significant terms from the 2-way PERMANOVA analyses with fungal, ciliate or bacterial communities as dependent variables, and site and temperature as explanatory variables, with indication of the significant pairwise comparisons.

Dependent variable	Significant terms	d.f.	Pseudo-F	p-perm	Terms/levels of factor	p-perm
Fungi	site × temperature	2	1.9417	0.02	*no significant terms*	*ns*
	site	1	2.1485	0.02	sandflat *vs Zostera*	0.02
	temperature	2	1.7761	0.01	control *vs* no warmness	0.04
					control *vs* warmness	0.03
Ciliate	site × temperature	2	1.7003	0.03	*no significant terms*	*ns*
	site	1	2.956	0.002	sandflat *vs* Zostera	0.004
	temperature	2	1.6075	0.03	control *vs* no warmness	0.03
					control *vs* warmness	0.03
Bacteria	site × temperature	2	5.4363	0.001	*no significant terms*	*ns*
	site	1	2.8995	0.003	sandflat *vs Zostera*	0.004
	temperature	2	9.0145	0.001	control *vs* no warmness	0.003
					control *vs* warmness	0.002
					no warmness *vs* warmness	0.002

#### Biological process – particle reworking

Evidence that bioturbation was affected by the different levels of the tested factors was found. The minimal adequate models were GLS regressions (Table 4), with a variance structure of different spreads for each independent fixed factor (full structure of the models, correction of the random part, pairwise comparison matrix for significant differences among levels and graphic representation of model predictions in Supplementary Material - Model 1 to Model 4; mean values and standard errors for each measurement in Supplementary Table S1).

**Table 4:** Summary of significant terms from the GLS for particle reworking measurements, with bioturbation components as dependent variables and site, treatment and time as explanatory variables.

Dependent variable	Significant terms	d.f.	L-ratio	p-value
SBR	site × temperature × time	7	16.813	0.0186
^f-SPI^L_mean_	site × temperature × time	7	20.003	0.0056
^f-SPI^L_median_	site	1	8.153	0.0043
^f-SPI^L_max_	site × temperature × time	7	20.316	0.0049

Surface Boundary Roughness (SBR), ^f-SPI^L_mean_ and ^f-SPI^L_max_ were significantly affected by the interaction of all factors (site × temperature × time), while ^f-SPI^L_median_ only varied with site (Table 4, models structure for the four bioturbation components described in Supplementary Material).

**Figure 5:**
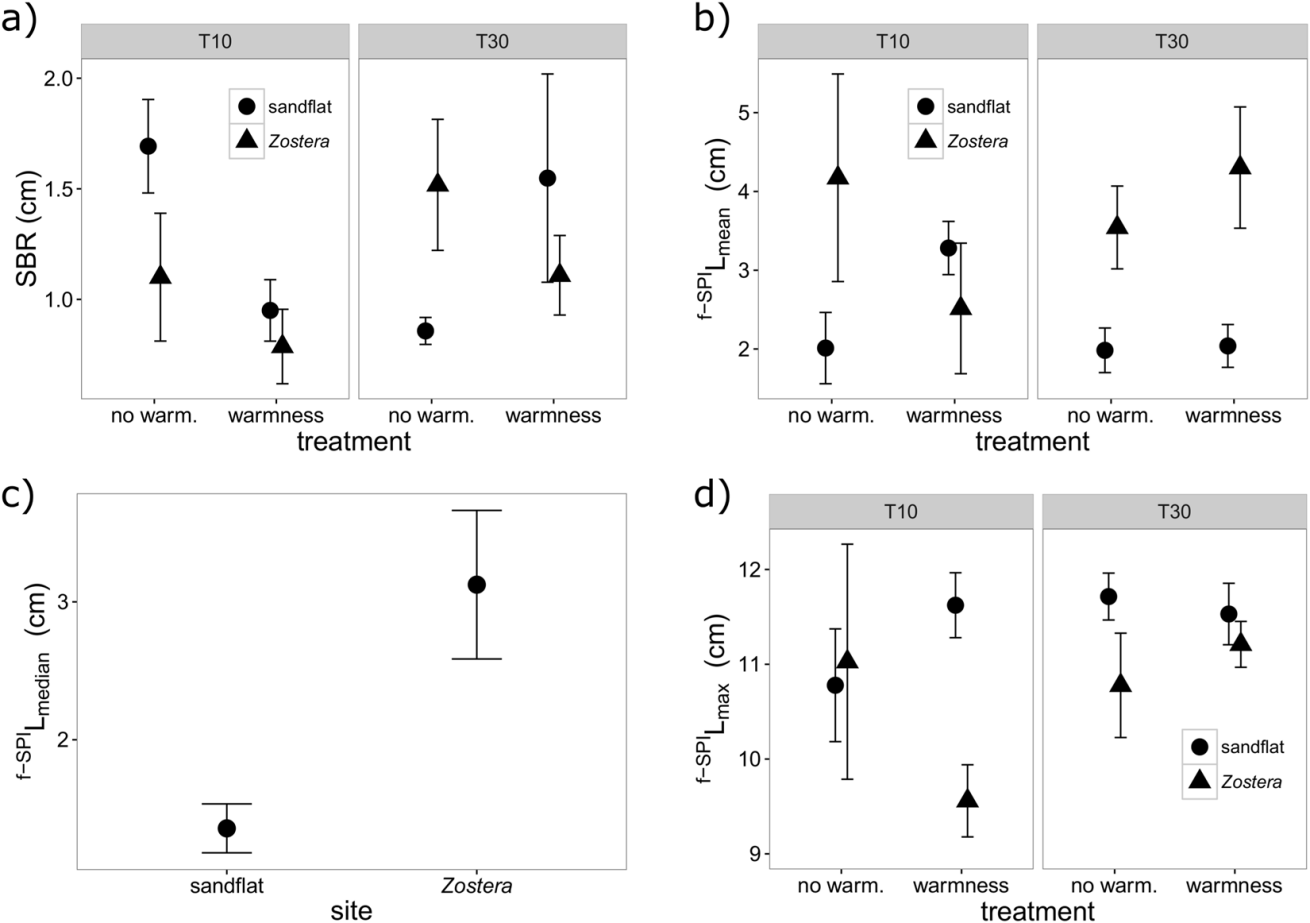
The significant effects of warmness treatment, site and time on surface boundary roughness (SBR) (**a**), ^f-SPI^L_mean_ (**b**), ^f-SPI^L_median_ (**c**) and ^f-SPI^L_mean_ (**d**) (cm, mean ± s.e.). For clarity, jitter has been applied to the x = argument of the plot function to avoid over-plotting.

^f-SPI^L_mean_ values ranged from 0.689 to 6.476 cm and reflected the complex 3-way interaction. At T10, ^f-SPI^L_mean_ values were lower with “no warmness effect” treatment and increased with the “warmness effect” for the sandflat (p = 0.0339, Supplementary Table S3), while the inverse tendency was found for the *Zostera* (Fig. 5b, Supplementary Table S1). At T30, in the *Zostera* bed, ^f-SPI^L_mean_ increased for the “warmness effect” compared to the “no warmness” one (Fig. 5b, Supplementary Table S1). In the sandflat, ^f-SPI^L_mean_ values decreased with time for “warmness effect” (p = 0.0199, Supplementary Table S3), while for the *Zostera* bed ^f-SPI^L_mean_ decreased with time for the “no warmness effect” treatment and increased with the “warmness effect”. Generally, *Zostera* bed had higher values of ^f-SPI^L_mean_ than the sandflat at T30, for both “no warmness effect” (p = 0.0175) and “warmness effect” (p = 0.0072, Supplementary Table S3).

^f-SPI^L_median_ only varied with site and values ranged from 0 to 6.949 cm (Fig. 5c). These were considerably higher for the *Zostera* bed than for the sandflat (p = 0.004, Supplementary Table S1).

SBR values ranged from 0.358 to 2.470 cm. There was always a decrease in SBR when comparing the effect of the warmness treatment, a result that was consistent for both sites and T10 (Fig. 5a, Supplementary Table S1). For T30, SBR increased with the “warmness effect” (Fig. 5a, Supplementary Table S1). SBR increased with time for both temperature treatments in the *Zostera* site. However, in the sandflat SBR decreased with time for the “no warmness effect” treatment and increased with time for the “warmness effect” treatment, in agreement with the significant interaction detected. Despite the observed tendencies, when comparing relevant significant differences (i.e. comparisons of treatments that share the same levels of two different factors), those were only found between the “no warmness” vs “warmness” effects in the sandflat at T10 (p = 0.0072), between T10 and T30 in the sandflat under the “no warmness effect” (p = 0.0009) and between sites at T30 under the “no warmness effect” (p = 0.039) (Supplementary Table S2).

Values of ^f-SPI^L_max_ ranged from 8.561 to 12.490 cm and again reflected the complex 3-way interaction. Overall, there was a tendency for ^f-SPI^L_max_ to increase with time, both in the sandflat and in the *Zostera* bed (Fig. 5d). In the sandflat the increase was higher in the “no warmness effect” treatment than in the “warmness effect” treatment that showed a circumstantial decrease (Fig. 5d, Supplementary Table S1). In the *Zostera* bed the larger increase over time was found in the “warmness effect” treatment (relevant significant differences only detected for this scenario, p = 0.0027, Supplementary Table S4), with a small decrease in the “no warmness effect” treatment (Fig. 5d, Supplementary Table S1). When comparing temperature treatments, the “warmness effect” showed higher or similar values to the “no warmness effect”, except at T10, for the *Zostera* bed (Fig. 5d). Nevertheless, relevant significant differences were only found between the sandflat and *Zostera* bed, at T10, under the “warmness effect” (p = 0.0001, Supplementary Table S4).

#### Sediment OM, C and N pools and intertidal water nutrient concentrations

The average organic matter content in the sediments was 1.96 ± 0.02% (mean ± SD) in sandflat and 2.58 ± 0.02% in *Zostera*, respectively, with significant differences between sites (matched-pairs t-test = −206.621, df = 1, p-value = 0.003). Regarding C and N pools in the sediment at the beginning of the experiment, differences were observed between sites, with higher carbon and nitrogen concentrations observed in the *Zostera* meadows, as a result of the higher organic matter content (C [mean ± s.d]: 1.138 ± 0.25 mg L^−1^ in sandflat and 1.74 ± 0.36 mg L^−1^ in *Zostera;* N: 0.086 ± 0.027 mg L^−1^ in sandflat and 0.135 ± 0.027 mg L^−1^ in *Zostera*). However, these distinct sediment characteristics and distinct plant coverage were not reflected in the dissolved nutrient concentrations in the shallow low water pools. Overall, the range of nutrient concentrations at the sampling sites and time were within the described for the system^30^, taking into account tidal and circadian cycles. However, no distinct pattern was observed in the nitrogen and phosphorus concentrations of the shallow intertidal pools for all treatments, sites and times. Concentrations of dissolved inorganic nitrogen ranged from 0.005 to 0.58 mg L^−1^, with most values <0.25 mg L^−1^, while PO_4_-P concentrations ranged between 0.02 and 0.15 mg L^−1^, but most values were <0.05 mg L^−1^.

## Discussion

### The importance of *in situ* experiments in the context of global warming studies

The use of field experiments to assess the effects of biodiversity on ecosystem functioning could be complemented with laboratorial work^31, 32^ in order to fine tune the potential causal-effect relationship. Aquatic ecosystems are highly complex systems where organisms are challenged by other biological components, as well as by abiotic influences, with several levels of interactions. This is particularly true for estuaries, where environmental variables shift over several cycles^33, 34^. Therefore, the study of transitional systems in laboratory has challenges that are inherent to that complexity. Despite the high level of accuracy allowed by laboratorial work, *in situ* experiments provide a more realistic interpretation of natural processes that actually occur in the system. Nevertheless, *in situ* experiments face limitations concerning universality (e.g. refs 21 and 35): the outcomes can be site specific as context modulates biotic responses, either due to physiological events or behavioural alteration. Simultaneously, climate changes, including global warming, may have very specific local responses. Therefore, it is of great interest to compile data from a wide geographic range in order to better understand climate change consequences. The present work brings some insights on these possible consequences on an estuarine system, in particular regarding a temperature increase, that is within the range recently preconized by the Paris United Nations Conference on Climate Change −1.5 to 2 °C. Nevertheless, this temperature increase should be regarded as an ambitious goal, fairly more optimistic than most of global climatic models predictions^26^.

### The temperature increase effect on different habitats and in benthic communities

A more complete understanding of the consequences of warmness in benthic estuarine communities could be provided by looking upon the meiofauna, as they play a key role linking micro- and macrofauna. Nevertheless, in such a complex and detailed *in situ* approach, already involving a large multidisciplinary team and limited funding, we realized that all fauna communities could not be examined. Moreover, a previous study in the same location (the Mondego estuary)^36^, which followed the organic matter mineralization and nutrient dynamics (including denitrification) mediated by the three faunal groups found that microfauna was responsible for the majority of the ecosystem processes (67%), followed by macrofauna (18%) and to a lesser extent by meiofauna (7%). Consequentially, we addressed the microfauna, as it is known to be the most reactive faunal community to environmental change, and the macrofauna, which shows a slower response to external pressures.

In our study, temperature was measured on the top layers of the sediment, suggesting that the air inside the closed boxes (warmness effect) could be warmer than in the open boxes. This temperature differential could emulate plausible values found during heat wave events, despite the fact that the experimental procedures were conducted during fairly routine summer period. The temperature increase in the sediment was within the range of the acceptable temperature increase agreed at the Paris Conference^29^. Although the temperature difference between the treatments was not constant, due to night temperature and high tides periods, we found a general 1.5 to 2 °C increase for the warmness effect treatment during the daily peak measurements. Even so, the *Zostera* bed seems to be acting as a temperature buffer, since temperature range was generally lower than in the bare sandflat. Three different mechanisms could explain this result: 1) a shading effect that the *Zostera’* leaves produce on the underlying sediment, which could reduce the amount of sunlight that reaches the sediment and thus warming; 2) a different colour between the *Zostera* bed and the sandflat areas, since the sandflat has darker shade and, therefore, better efficiency in light absorption, becoming warmer than the *Zostera* bed; 3) a differential thermal buffer due to distinct water retention capacities provided by the seagrass leaves and rhizomes.

Regarding the benthic communities, a differential temperature regime could limit or stimulate some natural processes and cause shifts on the community structure and functioning associated with those communities^3, 37, 38^. However, our 1.5 to 2 °C temperature increase seemed less influential than initially expected. In fact, the differences between sites (*Zostera* bed and sandflat) were more evident than the differences between temperature treatments, for both the macrofauna and microbiological communities. Against expectations, our macrofauna diversity results were inconsistent with previous studies dealing with the effects of the temperature rise, which have reported increased mortalities^37, 38^ and decreased diversity^13^ under such scenario. Our temperature increase was lower than the considered in those studies, and diversity was sometimes higher in the warmness effect treatment than in the control and non-warmness ones. Similarly, the period during which the communities were exposed to the heating effect (10 days or 30 days) did not have a consistent/clear effect, except for a higher variability among treatments in the sandflat, particularly at T10. In other words, there was less variation in diversity indices among treatments for the *Zostera* bed. Still, when examining the composition and structure of the macrofauna communities, some differences emerged due to the temperature increase (warmness effect), particularly for the biomass levels in the sandflat, the area where the temperature increase was higher. Again, these results suggest that the *Zostera* bed may mitigate the temperature increase effect on the macrobenthic community, and that time may have allowed communities to adjust to the disturbance (e.g. under an acclimation process^39^).

The microfauna communities, analyzed only at T10, were slightly affected by the warmness treatments, similarly to the results observed for the macrofauna. In general, fungal and ciliate richness was higher in sandflat than in the *Zostera*. Under the temperature increase scenario, fungal communities declined in both areas while the ciliate ones decreased only in the sandflat. Significant differences were also detected for the structure of those communities regarding site and temperature. However, these differences were not clear in PCO plots, and whose variability explained was considerably low (<25.1%). On the other hand, bacterial diversity did not respond clearly to the treatments or site. However, the differences on the bacterial community structure were more expressive than fungal and ciliate communities: there were also significant differences with site and temperature and samples clustered taking into account these differences, particularly regarding the control and the other treatments. Overall, it seems that the warmness treatment had a larger impact in the microbial diversity than in the macrobenthic diversity. This was expected, since microfauna communities respond much faster to external factors than the macrofauna ones^40, 41^, especially due to faster generation times^42^. There is also the possibility that the macrofauna under the warmness stimulus could interfere with microbial communities by competitive interaction^43^. Despite the observed differences, in light of our initial question, how a 1.5 to 2 °C temperature increase would affect benthic communities, the variation trends obtained were not very expressive. Also, daily environmental fluctuations could conceal the influence of the warmness treatment on microbial communities, by diluting the differential temperature between treatments.

### Consequences on ecosystem processes and functions

Our next question was whether the changes in the benthic communities due to the warming would reflect in changes on the processes and functions that are usually associated with those communities. However, since differences in the benthic communities with regard to the temperature rise effect were not particularly consistent, we expect the same pattern to occur with these processes and functions. For almost all components of bioturbation, there were significant interactions between all factors, suggesting that temperature had an effect on bioturbation that depended on the site and sampling time. We used smaller sized corers when compared with other studies (usually width >10 cm: e.g. refs 19, 22 and 44). However, we were able to measure the general tendency in fauna movements because we evaluated the luminophores’ statistical distribution along an unidimensional direction and not taking into account particular features of biogenic structures^19, 45, 46^. Also, bioturbation is mediated by organisms within a large range of sizes^47, 48^, which implies that even if larger animals are kept outside the corers, particle movement was still detected. Surface boundary roughness (SBR) increased with temperature only for the sandflat and for the longer time (T30), which could be related with a temperature gradient decreasing from the sediment top downwards that stimulates some activity in the top layer of the sediment. The temperature increase on the top sediment layers was expected to trigger the downwards movement of animals. In a scenario of warmness, the same would happen, even if deeper sediment layers were able to buffer the overlaying temperature increase. The warmness treatment had no significant differences in ^f-SPI^L_med_ (Median Luminophore depth), which reflects the short-term depth of mixing^20^ and could be a good proxy for the avoidance/downwards movement behaviour. Differential values of ^f-SPI^L_med_ between treatments may be a consequence of differential heat avoidance behaviour by the infaunal organisms, although this was not the case in our experiment. Nevertheless, for the other conditions, the variation was the inverse and not conclusive with regard to the temperature rise effects. For the other components of bioturbation, the variation tendency was also not clear or conclusive with regard to the temperature increase. We expected an overall bioturbation modification with temperature, as species respond differently to thermal pressures^14, 44^. However, the interaction of several factors (including those not assessed in the experiment, e.g. salinity, water flow, turbidity) resulted in indistinct tendencies, with large variation in the sediment mixing measurements within each treatment replicate. In fact, most of the studied items in this experiment showed a similar outcome: the 1.5–2 °C temperature increase had a smaller influence than site (i.e., with or without vegetation).

The absence of effects as a result of the temperature increase was also visible in the nutrient dynamics from the intertidal pools, at least at the specific sampling date and time. Despite the distinct sediment characteristics between sites, due to the presence of vegetation, effects on the sediment and shallow low-water pools nutrient fluxes due to warmness treatments were not perceptible. Data on the nutrients concentrations from the low water pools presented high variability, but were within the range of previously observed values for the system^49–51^. Due to the daily variation in physicochemical parameters in this shallow mesotidal system, which can be higher than seasonal variations^49, 50^, to detect consistent differences between the temperature treatments, 24 hours cycles would need to be performed, which was out of the scope of this research. Also, the great intraspecific variability observed in bioturbation was reflected in the dissolved nutrient concentrations, and may partially obscure possible effects of the temperature rise in the nutrient mineralization. Additionally, the tidal flushing and renewal of sampled intertidal pools may have hampered the evaluation of cumulative effects derived from temperature rise. Intra-treatment variability was considerable in some cases, reflecting the inherent heterogeneity of the system, and may have masked possible differences between warmness treatments. The same rationale may justify the absence of coherent differences between the sandflat and *Zostera* sites, despite the different nutrient pools observed in the sediment. Overall, our findings suggest that the 1.5–2 °C temperature rise will not significantly affect the nutrient biogeochemistry of this mesotidal system, as well as it will not force a clear shift in the ecosystem process of bioturbation.

### Is the system able to cope with 2 °C temperature increase?

Dramatic shifts in diversity and in the rate of processes and functions with environmental disturbance are more discernible when organisms are living in the edge of their vital tolerance range^52, 53^. Most of the organisms found in this experiment are probably well adapted to mild changes in the temperature, as part of their tolerance/ability to cope with the highly dynamic nature of the estuarine system. Also, acclimation events may occur^39, 54^, allowing organisms to better cope with a temperature increase. The fact that at T30 the macrobenthic diversity indices became more homogenous between treatments may be another indication of this acclimation process. In this study, site was more important than all other factors in the mediation of responses of the assessed biological and ecological components. An ecosystem relies on each of its components, either biological or environmental, as well as on its spatial heterogeneity to maintain its stability. Therefore, the diversity of habitats may enhance the overall resistance and resilience of an ecosystem^53, 55, 56^. One key finding of the present experiment is that different habitats respond differently to similar disturbances and that the number of possible interactions occurring in natural systems (among organisms and/or between organisms and their environment) may conceal the effects of mild disturbances. Also, our results emphasize the context dependency of the ecological responses to global changes. We acknowledge that the option for the small experimental plots is a trade-off between a possible way to induce the warmness effect and a reduced impact in the overall ecosystem, which is under recovery after some management efforts^30, 51^. Nevertheless, the results of our experiment seemed to reflect some of the consequences of the temperature increase suggested by the Paris Conference on Climate Change. Taking into account all the measured components, we may conclude that this estuarine system will, most probably, be able to cope with the temperature increase that is preconized as a global goal by recent international agreements.

## Material and Methods

### Study area

Field experiments were conducted during the summer of 2014 in the Mondego estuary. This is a relatively small mesotidal system with 8.6 km^2^, located in a warm temperate region at the western-Atlantic coast of Portugal (40°08′N, 8°50′W). The experimental set up took place in a sandflat area located in the inner part of the estuary (Supplementary Fig. S6). This area is currently characterized by low water flow (0.8–1.2 m.s^−1^) and fine sand sediments (median grain size according to the Folk and Ward Method^57^). Currently, more than 2/3 of the intertidal area is covered by the seagrass *Zostera noltei*, with bare sediments in the remaining area. Experimental plots were assembled within a close distance, ensuring that external abiotic pressures were fairly similar among plots (max. distance between plots: ~40 m). The emersion and immersion periods were the same for all the plots as they were at the same height. This allowed investigating the responses of benthic communities from different sites (with or without the seagrass) under air temperature warming simulation conditions.

### Experimental set-up

Two sites/habitat types were compared: *Zostera noltei* bed and adjacent sandflat. Transparent plastic boxes (57 × 39 × 28 cm) were used in the experiment, thus creating a warmness effect by enabling the penetration of sunlight and preventing heat dissipation. Changes in the light regime were not expected inside the boxes. They were placed in the sediment buried until half of its height (15 cm approximately). Tidal water had free circulation through small holes drilled in the larger sides of the boxes (8 mm Ø, disposed in two horizontal lines with 12 holes each right above the sediment surface). Temperature at the top layers of the sediment was monitored in both sites at intervals of 10 minutes during 30 days by Onset® HOBO Water Temperature Pro v2 Data Loggers. Three treatments with three replicates each were run in each site: 1) control without the box; 2) control box open (allowing heat dissipation, with a mesh (1 cm mesh size) at the top to avoid disturbance/predation by crabs and shorebirds, therefore keeping the “cage” effect), henceforward referred as “no warmness effect”; 3) box closed, referred as “warmness effect”.

Two sampling moments were selected (T10 – after 10 days; T30 – after 30 days) to observe potential short- and long-term effects of warmness in the benthic communities of both sites. A set of experimental plots were assembled for each sampling moment. After each sampling occasion, the respective plots were disassembled. For each sampling time and site, sediment cores (141 cm^2^ surface area), for benthic communities, were collected after removing the boxes (total of 36 samples: 3 replicates for 2 sites, 3 experimental treatments and 2 sampling times). These samples were washed in estuarine water through a 500 μm mesh sieve and the benthic organisms retained were preserved in 4% buffered formalin. Afterwards, in the laboratory, animals were sorted and transferred to 70% ethanol, identified to the lowest possible taxon and counted. The ash-free dry weight (AFDW; 8 h at 450 °C) was assessed. Additional samples were taken at T10 with a 26 mm diameter core, from the superficial layers (up to 3 cm depth), for microbiological community characterization. At each sampling occasion and for each treatment, three water aliquots (20 mL) from intertidal pools (outside and inside the boxes; please notice that some of these intertidal pools can have small areas) and three surface sediment aliquots (up to 3 cm depth) were taken for nutrient analyses (described in detail in the following sections).

### Microbial diversity

DNA was extracted from 250 mg of freeze-dried sediment using the PowerSoil® DNA Isolation Kit (MoBio Laboratories, Solana Beach, CA, USA) according to the manufacturer’s instructions. The ITS2 region of fungal ribosomal DNA (rDNA) was amplified with the primer pair ITS3GC and ITS4, the V3 region of 16S bacterial rDNA was amplified with the primer pair 338GC and 518^58^, and the 18S rDNA of ciliates was amplified with the primer pair 384GC and 1147^59^.

For PCR of fungal, bacterial and ciliate DNA, 1x Green GoTaq® Flexi Buffer, 3 mM of MgCl2, 0.2 mM of dNTPs, 0.4 μM of the appropriate primers, 1.5 U of GoTaq® G2 Flexi DNA Polymerase (Promega) and 2 μL of DNA were used in a final volume of 50 μL. PCRs were carried out in a MyCycler Thermal Cycler (BioRad Laboratories, Hercules, CA, USA). The PCR program for bacteria and fungi was initial denaturation at 95 °C for 2 min, 36 cycles of denaturation at 95 °C for 30 s, primer annealing at 55 °C for 30 s and extension at 72 °C for 1 min, and final extension at 72 °C for 30 min (modified from ref. 58). The PCR program for ciliates was initial denaturation at 94 °C for 5 min, 30 cycles of denaturation at 94 °C for 45 s, primer annealing at 55 °C for 60 s and extension at 72 °C for 90 s, and final extension at 72 °C for 7 min^59, 60^.

DGGE (Denaturing Gradient Gel Electrophoresis) analysis was performed using a DCodeTM Universal Mutation Detection System^58^ (BioRad Laboratories, Hercules, CA, USA). For fungi, 700 ng of the amplified DNA products with 380–400 bp was loaded on 8% (w/v) polyacrylamide gel in 1− Tris–acetate–EDTA (TAE 1×) with a denaturing gradient from 30 to 60% (100% denaturant corresponds to 40% formamide and 7 M urea). For bacteria, 700 ng of the amplified DNA products of ca. 200 bp was loaded on 8% (w/v) polyacrylamide gel in 1 × TAE with a denaturing gradient from 45 to 62.5%. For ciliates, 700 ng of the amplified DNA products with 750–800 bp was loaded on 6% (w/v) polyacrylamide gel in 1 × TAE with a denaturing gradient from 20 to 42.5%. Fungal and bacterial DNA was separated at 55 V and 56 °C, while ciliate DNA was separated at 80 V and 60 °C. All gels were run for 16 h. Gels were stained with 1x Midori Green (NIPPON Genetics EUROPE GmbH, Düren, Germany) for 10 min and gel images were captured under UV light in a gel documentation system (ChemiDoc XRS, BioRad).

### Measurement of particle reworking *in situ*

Particle reworking of the sediment (i.e. bioturbation) of each experimental unit was assessed using fluorescent sediment profile imaging (f-SPI^61^) a non-invasive method that allows tracking fluorescent-dyed sediment particles that shine under UV light (luminophores: 125–250 µm diameter, green colour; Brian Clegg, Ltd, UK)^62^. The distribution of luminophores can be determined from high spatial resolution images from the sides of a transparent corer.

For each test box, a square section corer with open top and bottom (clear PET plastic bottle, with the bottom removed; side 7 cm) was buried to a depth of approximately 15 cm. Luminophores (aprox. 20 g corer^−1^) were added at the beginning of the experiment. At T10 and T30 the corers were retrieved by hermetically covering the top side (creating negative pressure). After being cleaned, the squared corers were photographed on all four sides within a dark box with UV illumination. We used a Canon EOS 350D single lens reflex digital CMOS camera (8.0 megapixels; exposure 4 s, f = 4 and a film equivalent speed of ISO 100). The resulting images (red-green-blue [RGB] colour with JPEG [Joint Photographic Experts Group] compression) were cropped to the full width of the corer and all four sides were merged in one image (2188 pixels, effective resolution = 12.8 µm per pixel). These images were analyzed using a custom-made plugin that runs within ImageJ (Version 1.48c), a java-based public domain program developed at the US National Institute of Health (available at http://imagej.nih.gov/ij/). This plugin assessed the values of mean (^f-SPI^L_mean_, time dependent indication of mixing), median (^f-SPI^L_med_, typical short-term depth of mixing) and maximum (^f-SPI^L_max_, maximum extent of mixing over the long-term) mixed depths of particle redistribution^20^. Surface Boundary Roughness (SBR), the maximum vertical deviation of the sediment-water interface, (upper – lower limit) was also measured, as an indication of surficial activity.

### Sediment OM, C and N pools and dissolved inorganic nutrients

Sediment aliquots (three replicates per site, treatment, T0 and after 30 days) were sampled, homogenised and analysed for organic matter (OM) content through loss on ignition (LOI%; 6 h combustion at 500 °C), and for total Carbon (TC) and total Nitrogen (TN) in a CHNS/O analyser (Fisons Instruments Model EA 1108, Beverly, Massachusetts, USA).

A total of 36 water aliquots (three replicates per site, treatment and sampling time) were sampled and analysed for dissolved inorganic nitrogen (DIN = NH_4_-N + NO_x_-N) and dissolved inorganic phosphorous (DIP = PO_4_-P). Immediately after sampling, water aliquots (20 mL) were transported to the laboratory in cool boxes (dark and 4 °C), where they were filtered (0.7 μm glass-fibre filter, Whatman GF/F) and stored frozen at −18 °C until analysis. Concentrations of nitrite (NO_2_-N) and nitrate (NO_3_-N) were determined using a flow injection system (FIAstar 5000 Analyzer, Höganäs, Sweden), following the Strickland and Parsons^63^ method. The determination of the concentrations of ammonium (NH_4_-N) and phosphate (PO_4_-P) was done following the standard method described in Limnologisk Metodik^64^. To ensure the analytical quality control, calibration curves, using a standard solution, were run at the beginning of the analysis and in parallel with blanks and samples.

### Data analysis

Species richness (number of species) and Shannon-Wiener diversity were computed for the macrofauna communities. These indices and benthic community data were treated with Permutational Multivariate Analyses of Variance – PERMANOVA, to test potential differences with regard to the temperature treatment, sites and time. PERMANOVA is an analysis of variance to test one or more factors, using permutation methods and on the basis of a resemblance matrix^65^. PERMANOVA was carried out on three-factor crossed experimental design with replication for the diversity indices, upon Euclidean distance matrix for non-transformed data, and for benthic community, upon a Bray-Curtis similarity matrix, with a dummy variable to compensate for zero occurrences^65^. The explanatory factors were included as fixed factors and three levels for temperature treatment (control; no warmness effect; warmness effect), two levels for time (T10 and T30) and two for site (sandflat and *Zostera*) were considered. The benthic data were further explored using Principal Coordinates Analyses (PCO), where we overlaid vectors based on Spearman correlations onto the PCO plot in order to clarify patterns of change^65^.

For microfauna, DGGE gels were aligned and normalized using BioNumerics 7.1 (Applied Maths, Sint-Martens-Latem, Belgium). Each DGGE band was considered one operational taxonomic unit (OTU), taking into account that more than one species can co-migrate to the same position in the gel. Species richness was assessed as the number of OTUs and the Shannon-Wiener diversity computed for all microfauna communities (fungi, ciliates and bacteria). The differences in diversity were tested with a two-way ANOVA for the effects of site and temperature treatment (there was only one sampling occasion for microfauna: T10), followed by a Tukey’s post-hoc test^66^. Assumptions of the ANOVA were initially verified. Bacterial richness was Box-Cox-transformed to achieve normality. Statistical analyses were done in STATISTICA (version 8.0 for Windows; Statsoft, Tulsa, Oklahoma). We used 2-way PERMANOVA to test the effect of site and temperature on the aquatic microfauna assemblages (based on DGGE fingerprints)^65^. Prior to the analyses, data was (square root) √(x)-transformed and converted into a Bray–Curtis similarity matrix. All diversity and community data analyses for the macrofauna and microfauna were performed using PRIMER v6 software with the PERMANOVA add-on package^65^.

For particle reworking analyses, we developed independent regression models for each of our dependent variables (SBR, ^f-SPI^L_mean_, ^f-SPI^L_med_, ^f-SPI^L_max_) using the full factorial combination of independent variables (site [sandflat; *Zostera*], temperature treatment [no warmness effect; warmness effect] and time [T10; T30]). The “no warmness effect” worked as control for this analysis. A generalised least squares (GLS^67^) estimation procedure was used and included the appropriate variance-covariate structure (Minimal adequate models structure in Supplementary Material), as data violated the homoscedasticity assumption. GLS regression allows the residual spread to vary with the explanatory variables and avoids data transformation. Restricted maximum-likelihood (REML) estimation was used to determine the optimal variance-covariate structure, after finding the lowest Akaike Information Criteria (AIC) and best model residuals. The optimal fixed structure was then determined by backward selection using the likelihood ratio (L-ratio) test obtained using maximum likelihood (ML) estimation and the minimal adequate model was re-expressed using REML^68–70^. We used a parametric bootstrap with 999 re-samples and the percentile method to obtain the 95% CI limits around the predicted values (Supplementary Material, Models 1–4). GLS analyses were conducted using the ‘nlme’ package^71^ and parametric bootstrapping were conducted, assuming that the estimated parameters followed a multivariate Gaussian distribution with mean and variances provided from the output of the fitting function, using the function ‘rmvnorm’ within the package ‘mvtnorm’^72^. These analyses were performed using the ‘R’ statistical and programming environment (R Development Core Team 2012).

## Electronic supplementary material

Supplementary Material
