# Supplementary material for: New climatic targets against global warming: will the maximum 2 °C temperature rise affect estuarine benthic communities?
Source: Sci Rep. 2017 Jun 20;7:3918. doi: 10.1038/s41598-017-04309-0 (PMC5478632; doi:10.1038/s41598-017-04309-0)
Supplement: Supplementary file 1 — Supplementary Material [file 41598_2017_4309_MOESM1_ESM.pdf]

Supplementary Material

New climatic targets against global warming: will the maximum 2ºC temperature rise affect estuarine benthic communities?

Daniel Crespo*, Tiago Fernandes Grilo, Joana Baptista, João Pedro Coelho, Ana Isabel Lillebø, Fernanda Cássio, Inês Fernandes, Cláudia Pascoal, Miguel Ângelo Pardal, Marina Dolbeth

* Corresponding author:

Centre for Functional Ecology – CFE, Department of Life Sciences, University of Coimbra

Calçada Martim de Freitas, 3000-456 Coimbra, Portugal

e-mail: daniel.crespo@uc.pt; Tel: +351 239 855 760 (Ext. 262264)

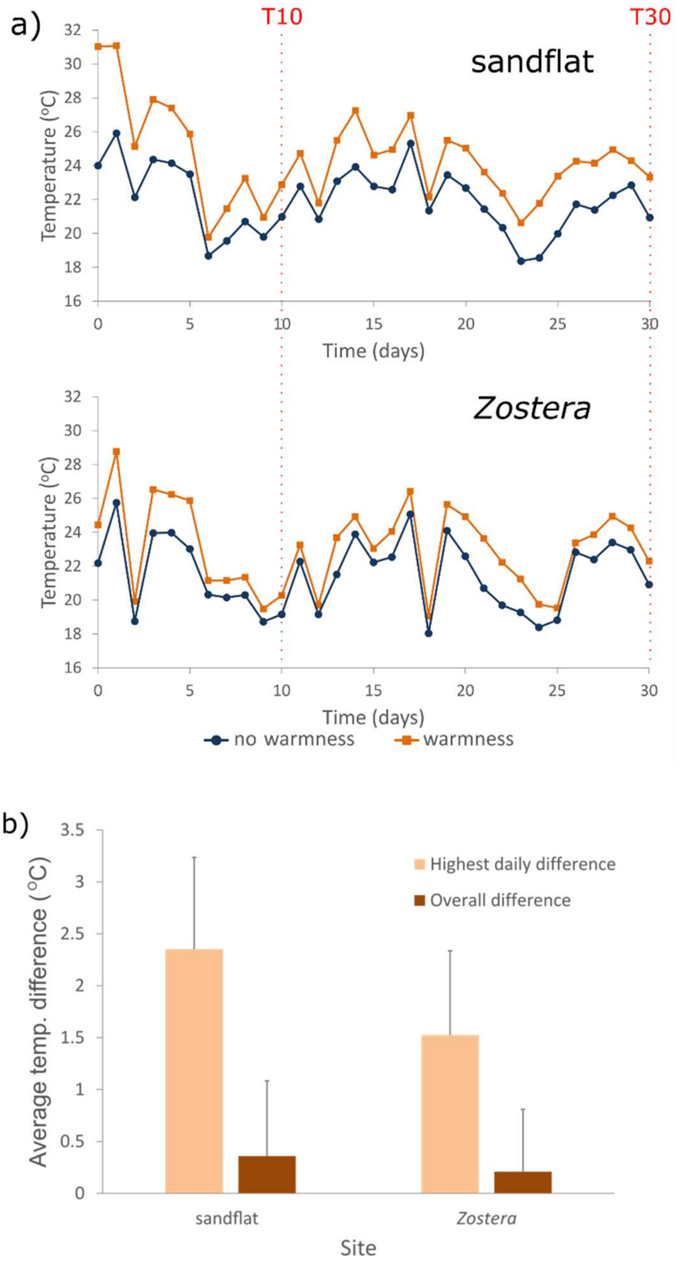

Supplementary Figure S1. Temperature registered during the experiment for "no warmness effect" (box open, acting as control for the effect of the box) and "warmness effect" (box closed) and for both sites: (a) highest daily temperature, with indication of the sampling periods, after 10 days (T10) and after 30 days (T30); (b) average difference and standard deviation within treatments in both sites. The highest daily difference is the average difference for every daily heat peak and the overall difference refers to all temperature records (including high tides and night periods).

Structure of the minimal adequate models for particle reworking, with site, treatment and time as explanatory variables

Supplementary Table S1. Mean values and standard error for the particle reworking measurements, for significant levels of each factor on the minimal adequate models.

**variable**			SBR		$^{f\text{-}SPI}L_{mean}$		$^{f\text{-}SPI}L_{median}$		$^{f\text{-}SPI}L_{max}$	
site	time	temperature treatment	Mean	± S.E.	Mean	± S.E.	Mean	± S.E.	Mean	± S.E.
sandflat	T10	no warm.	1.692	0.211	2.012	0.453	1.357	0.177	10.778	0.595
		warmn.	0.950	0.139	3.282	0.338			11.623	0.343
	T30	no warm.	0.857	0.061	1.983	0.283			11.714	0.247
		warmn.	1.548	0.471	2.039	0.272			11.530	0.325
Zostera	T10	no warm.	1.101	0.289	4.174	1.317	3.125	0.539	11.027	1.240
		warmn.	0.787	0.168	2.515	0.829			9.559	0.380
	T30	no warm.	1.518	0.296	3.543	0.526			10.778	0.551
		warmn.	1.109	0.180	4.303	0.769			11.210	0.242

MODEL 1 – Surface Boundary Roughness (SBR)

SBR = f (site x warmness treatment x time)

The model was a linear regression model with a GLS extension, with *time* as variance - covariate.

Supplementary Table S2. Pairwise comparison matrix for significant differences among levels for Surface Boundary Roughness. Significant differences are highlighted.

		time	T10				T30			
		site	sandflat		*Zostera*		sandflat		*Zostera*	
time	site	temperature treatment	no warm.	warm	no warm.	warm	no warm.	warm	no warm.	warm
T10	sandflat	no warm.		**.0072**	.1113	.3169	**.0009**	**.0135**	**.0133**	**.0448**
		warmn.			.3169	.4623	**.0135**	.2347	**.0448**	.6196
	Zostera	no warm.				.3580	**.0133**	**.0448**	.3235	.8451
		warmn.					**.0448**	.6196	.8451	.2035
T30	sandflat	no warm.						.1584	**.0390**	.0735
		warmn.							.1584	.3919
	Zostera	no warm.								.2497
		warmn.								

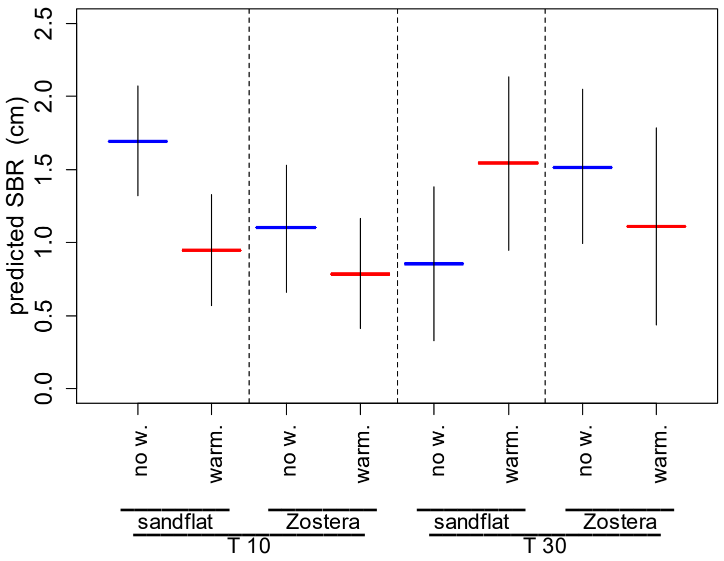

Supplementary Figure S2. Predictions of the minimal adequate regression model (GLS extension with *time* as variance – covariate) for the Surface Boundary Roughness for two different temperature treatments, in two different sites, at two sapling moments: horizontal bars represent the predicted values from the minimal adequate regression model (n = 32) and the vertical lines represent the confidence intervals (± 95 %).

MODEL 2 – f-SPI Luminophore Mean Depth ($^{f\text{-}SPI} L_{mean}$)

$^{f\text{-}SPI} L_{mean}$ = f (site x treatment x time)

The model was a linear regression model with a GLS extension, with *site* as variance - covariate.

Supplementary Table S3. Pairwise comparison matrix for significant differences among levels for f-SPI Luminophore Mean Depth. Significant differences are highlighted.

		time	T10				T30			
		site	sandflat		*Zostera*		sandflat		*Zostera*	
time	site	temperature treatment	no warm.	warm	no warm.	warm	no warm.	warm	no warm.	warm
T10	sandflat	no warm.		**.0339**	.0804	.0761	.9532	.0921	.6556	.0626
		warmn.			.0761	.4702	.0921	**.0199**	.0626	**.0283**
	Zostera	no warm.				.272	.6556	.0626	.6168	.1743
		warmn.					.0626	**.0283**	.1743	.1489
T30	sandflat	no warm.						.8906	**.0175**	.4809
		warmn.							.4809	**.0072**
	Zostera	no warm.								.4060
		warmn.								

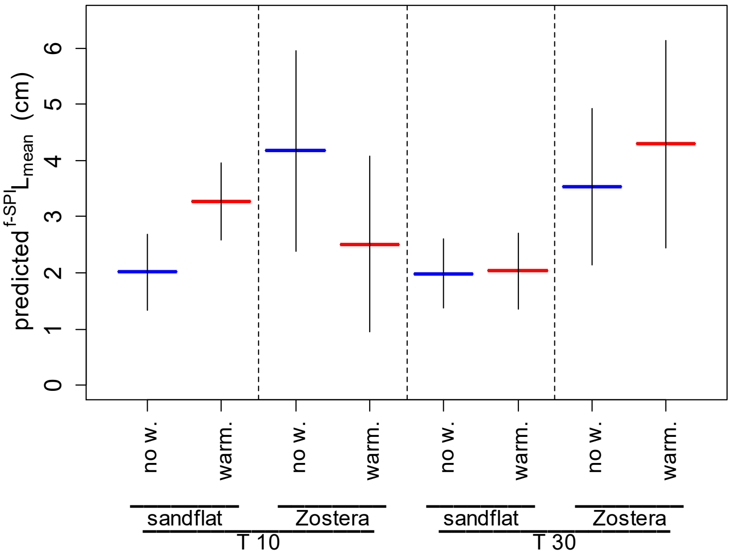

Supplementary Figure S3. Predictions of the minimal adequate regression model (GLS extension with *site* as variance – covariate) for the f-SPI Luminophore Mean Depth for two different temperature treatments, in two different sites, at two sapling moments: horizontal bars represent the predicted values from the minimal adequate regression model (n = 32) and the vertical lines represent the confidence intervals (± 95 %).

MODEL 3 – f-SPI Luminophore Median Depth ($^{f\text{-}SPI}L_{med}$)

$^{f\text{-}SPI}L_{median}$ = f (site)

The model was a linear regression model with a GLS extension, with *site* as variance - covariate. Sandflat vs *Zostera* bed: p= 0.004.

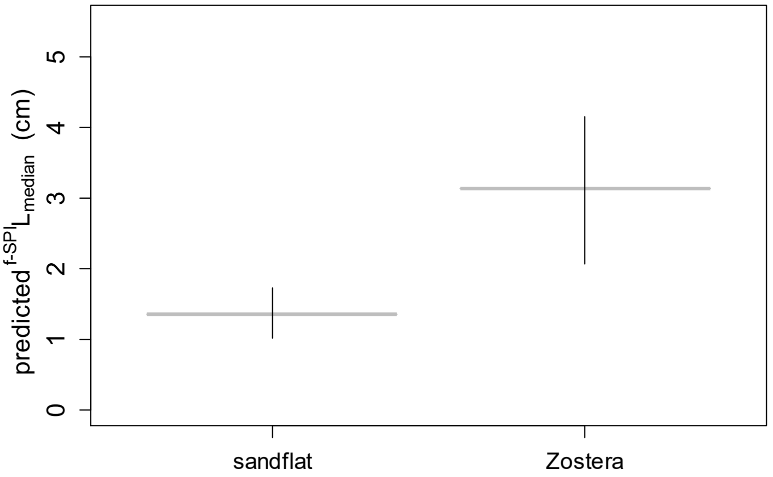

Supplementary Figure S4. Predictions of the minimal adequate regression model (GLS extension with *site* as variance – covariate) for the f-SPI Luminophore Median Depth for two different sites: horizontal bars represent the predicted values from the minimal adequate regression model (n = 32) and the vertical lines represent the confidence intervals (± 95 %).

MODEL 4 – f-SPI Luminophore Max Depth ($^{f\text{-}SPI}L_{max}$)

$^{f\text{-}SPI}L_{max}$ = f (site x treatment x time)

The model was a linear regression model with a GLS extension, with *treatment* as variance - covariate.

Supplementary Table S4. Pairwise comparison matrix for significant differences among levels for f-SPI Luminophore Max Depth. Significant differences are highlighted.

		time	T10				T30			
		site	sandflat		*Zostera*		sandflat		*Zostera*	
time	site	temperature treatment	no warm.	warm	no warm.	warm	no warm.	warm	no warm.	warm
T10	sandflat	no warm.		.2455	.7991	**.0408**	.2808	.3044	.3540	.0525
		warmn.			**.0408**	**.0001**	.3044	.8517	.0525	**.0194**
	Zostera	no warm.				.0786	.3540	.0525	.7896	.0821
		warmn.					.0525	**.0194**	.0821	**.0027**
T30	sandflat	no warm.						.7876	.2531	.5251
		warmn.							.5251	.5479
	Zostera	no warm.								.5286
		warmn.								

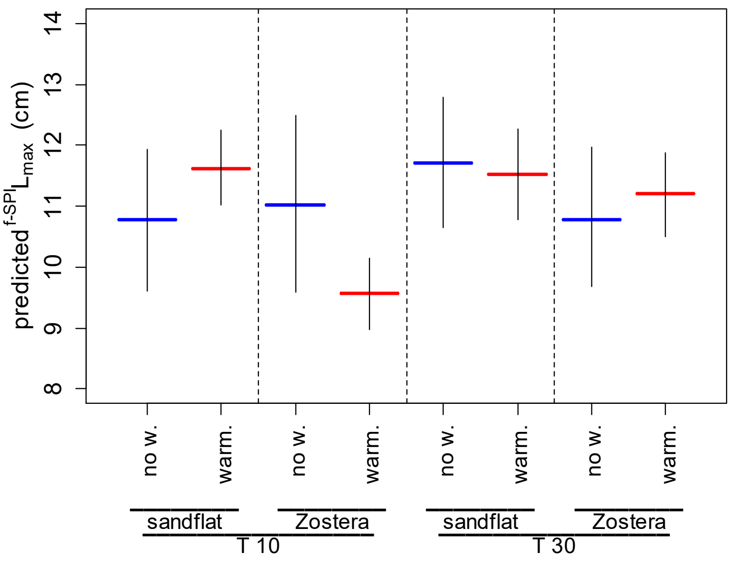

Supplementary Figure S5. Predictions of the minimal adequate regression model (GLS extension with *treatment* as variance – covariate) for the f-SPI Luminophore Maximum Depth for two different temperature treatments, in two different sites, at two sapling moments: horizontal bars represent the predicted values from the minimal adequate regression model (n = 32) and the vertical lines represent the confidence intervals (± 95 %).

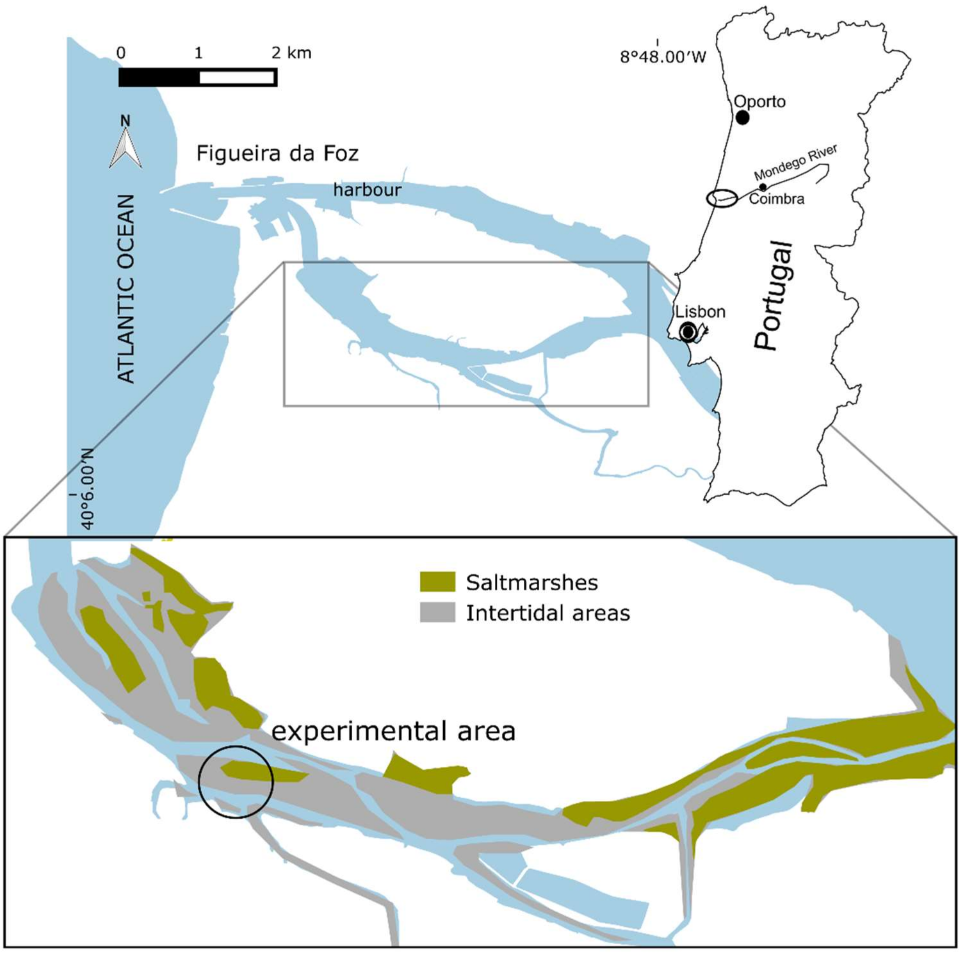

Supplementary Figure S6. The Mondego estuary, showing the experiment location in detail (map produced with the open source software QGIS 2.16.1-Nødebo, http://qgis.org, edited with the open source software Inkscape 0.91, http://www.inkscape.org).
